# XR-based bullying prevention interventions among K–12 students: a scoping review of design, theory, and outcomes

**DOI:** 10.3389/fpsyt.2026.1978530

**Published:** 2026-10-06

**Authors:** Xiayu Li, Heng Luo, Zi Wang, Lele Li

**Affiliations:** Faculty of Artificial Intelligence in Education, Central China Normal University, Wuhan, China

**Keywords:** augmented reality, bullying prevention, extended reality, K–12 education, scoping review, virtual reality

## Abstract

**Background:**

Extended reality (XR) can present controlled and repeatable bullying scenarios, support perspective and role changes, and provide opportunities for response practice. However, how XR affordances have been connected to prevention mechanisms, learning activities, and outcomes remains unclear.

**Objective:**

This scoping review mapped the characteristics of XR-based bullying prevention interventions for K–12 students, examined the alignment among theoretical mechanisms, XR affordances, and learning activities, and synthesized reported outcome patterns.

**Methods:**

Four databases (Web of Science, Scopus, ERIC, and PsycINFO) were searched on March 13, 2026, for peer-reviewed articles published from 2005 through 2025. Targeted supplementary searching was also conducted. Eligible articles evaluated direct prevention programs or XR applications explicitly developed to inform bullying prevention through mechanism testing, scenario validation, or feasibility evaluation. Data from 17 articles were charted and synthesized descriptively in accordance with PRISMA-ScR guidance.

**Results:**

Fifteen articles used virtual reality or virtual environments and two used augmented reality. Twelve used high-immersion configurations, but nine of these provided limited learner interactivity. The evidence base comprised nine program evaluations, five mechanism-oriented studies, and three design or feasibility studies. Immersion was most consistently associated with presence, realism, and immediate emotional responses. Favorable or conditionally favorable intervention-related evidence patterns were reported in several studies incorporating meaningful learner interaction, although these findings varied across outcomes, study designs, and evaluation conditions. Evidence for knowledge, empathy, attitudes, self-efficacy, and behavioral intentions was mixed, and only a small subset of studies examined real-world bullying-related behavior or outcomes beyond the immediate post-intervention period. Two recurring instructional patterns were identified: experience followed by reflection and action followed by feedback and revision.

**Conclusions:**

XR can make bullying situations salient, controllable, and experientially accessible, but stronger immersion should not be equated with stronger prevention effects. Future interventions should specify the intended mechanism, select XR affordances and learning activities accordingly, and evaluate outcomes at the level and timescale supported by the intervention design. Such alignment may clarify when XR provides meaningful prevention value beyond less immersive or non-digital alternatives.

## 1 Introduction

Bullying is commonly defined as intentional and repeated harm occurring within relationships characterized by a power imbalance (1). A recent meta-analysis of 116 studies involving 603,231 children and adolescents estimated the pooled prevalence of bullying victimization at approximately 25% (2). Its psychological and social consequences may persist into adulthood (3, 4). Bullying is also embedded in peer-group dynamics: students may act as assistants, reinforcers, defenders, or outsiders, and their responses can sustain or interrupt the process (5). Prevention therefore requires more than conveying rules. Students need opportunities to recognize bullying, interpret the roles and responsibilities of those involved, and practice appropriate responses.

School-based programs commonly use discussion, video, cooperative activities, and role-play to support these goals. KiVa, for example, combines classroom activities and role-play to strengthen empathy, understanding of peer-group processes, and strategies for supporting victims (6). Face-to-face enactment nevertheless brings practical and ethical constraints. Teachers may have limited time or experience with drama-based activities, and students may feel anxious, exposed, or vulnerable when publicly performing bullying roles (7, 8). Digitally simulated situations can make scenarios more controllable and repeatable while creating opportunities for learners to encounter perspectives and decisions that may be difficult to reproduce safely in a classroom.

In this review, extended reality (XR) is used operationally as an inclusive term for virtual reality (VR), augmented reality (AR), immersive 360° media, and interactive virtual environments that create, mediate, or simulate interactions between physical and digital environments. This definition accommodates the terminology and levels of immersion represented in an emerging and technically heterogeneous literature, consistent with earlier conceptualizations of augmented reality and the reality–virtuality continuum (9, 10). It does not imply that all included studies explicitly described their systems as XR. VR can surround learners with simulated bullying situations or present events from a first-person victim or bystander perspective; AR can overlay virtual characters and information onto physical settings; and desktop virtual environments can support repeated choices, feedback, and role-based problem solving without head-mounted displays. These configurations differ not only in sensory immersion but also in interactivity, learner agency, embodiment, role participation, and how instructional support is organized.

Previous reviews have examined overlapping portions of this literature. Boboc and Damaševičius (11) reviewed XR applications across bullying identification, prevention, and victimization for participants ranging from children to adults. Xue et al. (12) considered VR and AR for studying bystander behavior across multiple interpersonal-violence contexts. Serritella et al. (13) reviewed technology-supported bullying and cyberbullying interventions more broadly, while Sun et al. (14) synthesized the effectiveness of VR-based anti-bullying interventions. These reviews established the growing relevance of immersive technology, but they primarily focused on technology applications, intervention effectiveness, or broader violence-related contexts rather than examining how theoretical mechanisms, XR affordances, learning activities, and outcome measures were aligned within K–12 bullying prevention designs. Therefore, existing reviews provide limited guidance for understanding why similar XR technologies may produce different types of educational evidence across intervention designs and under what conditions particular XR features are expected to support bullying prevention processes.

This alignment matters because an immersive experience is not synonymous with an effective intervention. Presence, realism, or emotional arousal may indicate that a simulated event is compelling, but they do not by themselves explain whether or how learners develop new understandings, attitudes, response strategies, or behaviors. Similarly, a system may permit interaction without giving learners meaningful agency over feedback or subsequent events. Understanding these distinctions is particularly important for a scoping review because the evidence base remains small and heterogeneous in technology, purpose, design, and measurement (15, 16).

Accordingly, this review addressed three questions:

1. What are the study, technology, and intervention-design characteristics of XR-based bullying prevention interventions among K–12 students?
2. How have theoretical mechanisms, XR affordances, and learning activities been aligned?
3. What outcomes have been reported, and what evidence patterns, limitations, and gaps are apparent?

## 2 Methods

### 2.1 Review design and reporting framework

The review followed the five-stage framework proposed by Arksey and O’Malley (17): identifying the research questions, identifying relevant sources, selecting sources, charting data, and collating and reporting results. Updated JBI methodological guidance informed the formulation of eligibility criteria, data charting, and descriptive synthesis (16). Reporting was guided by the Preferred Reporting Items for Systematic Reviews and Meta-Analyses Extension for Scoping Reviews (PRISMA-ScR; 18). The review protocol was retrospectively registered with INPLASY (registration number: 202680060).

Because the purpose was to map the nature and configuration of an emerging evidence base rather than estimate a pooled intervention effect, no formal critical appraisal or risk-of-bias assessment was undertaken. Findings are therefore reported as patterns in the available literature and are not weighted by methodological quality.

### 2.2 Eligibility criteria

Eligibility was structured using the Population–Concept–Context framework. The population comprised students from kindergarten through Grade 12. The concept was an XR-based bullying prevention intervention. For this review, an intervention was defined as an XR-based activity, application, or program intentionally designed to prevent bullying or to support the development and evaluation of prevention through mechanism testing, scenario validation, or feasibility assessment. Studies that used XR only to observe or describe bullying-related phenomena, without an explicit prevention purpose, were excluded. This definition retained experimental-exposure and design studies when their stated purpose was to develop or test prevention-relevant experiences, but excluded XR research that neither addressed bullying-related phenomena nor contributed to prevention design. Closely connected phenomena such as peer victimization, social exclusion, cyberbullying, and peer sexual harassment were included when the article explicitly framed the XR application as preventive or as informing bystander, victim-support, or anti-bullying processes. No restriction was placed on the physical setting. Peer-reviewed empirical journal articles published from 2005 through 2025 were eligible. No formal language restriction was applied, provided sufficient information could be extracted. The detailed inclusion and exclusion criteria are listed in **Table 1**.

**Table 1:** Inclusion and exclusion criteria.

Domain	Inclusion criteria	Exclusion criteria
Population	K–12 students or studies including K–12 learners with extractable student-level data	Studies focusing exclusively on non-K–12 populations or without extractable K–12 student data
Concept	Direct XR-based bullying prevention programs or XR studies addressing prevention-related mechanisms, scenario validation, or feasibility	No XR component; no bullying-related phenomenon; no explicit prevention relevance
Context	Any educational or real-world setting involving K–12 bullying-related learning, prevention, or assessment	None based on physical setting
Evidence type	Empirical primary research reporting extractable methods and findings, including intervention, mechanism, validation, and feasibility studies	Reviews or meta-analysis, protocols, editorials, commentaries, and conceptual papers
Publication	Peer-reviewed journal articles published from 2005 through 2025	Conference abstracts or proceedings, dissertations or theses, books or book chapters, and other non-peer-reviewed sources

### 2.3 Information sources and search strategy

A systematic search was conducted on March 13, 2026 in Web of Science, Scopus, ERIC, and PsycINFO. Coverage was limited to publications dated January 1, 2005 through December 31, 2025. The search combined terms for bullying-related phenomena with terms for XR technologies. Targeted supplementary identification was undertaken through reference-list checking and web searching for named systems, programs, and related articles.

The Web of Science strategy was as follows: TS=[(bully* OR cyberbully* OR ‘school bullying’ OR ‘peer bullying’ OR ‘peer victimization’ OR ‘relational aggression’ OR ‘bystander behavior’ OR ‘sexual harassment’) AND (‘virtual reality’ OR VR OR ‘virtual environment*’ OR ‘immersive environment*’ OR ‘head-mounted display*’ OR HMD OR ‘extended reality’ OR XR OR ‘augmented reality’ OR AR OR ‘mixed reality’ OR MR OR simulation OR ‘virtual simulation’ OR ‘virtual world*’)]. Syntax was adapted to the other databases.

### 2.4 Source selection

Records were deduplicated before title and abstract screening. The study selection process included a preliminary topic screening step followed by formal screening and eligibility assessment. During the preliminary screening step, the first reviewer removed records that were clearly unrelated to the review scope or did not meet the predefined evidence-type or publication-type criteria. This step was conducted to improve screening efficiency by excluding obviously ineligible records before formal screening. During the formal screening stage, two reviewers independently conducted title and abstract screening and full-text assessment. Disagreements regarding study eligibility were discussed between the two reviewers until consensus was reached. To supplement the database search, the second author conducted citation-based searching of the included and closely related articles and screened the additionally identified records against the same eligibility criteria. Potentially eligible articles identified through this supplementary search were discussed by the review team before final inclusion. The database search identified 1,038 records. After removing 230 duplicates and 352 records excluded before screening based on predefined evidence-type and publication-type criteria, 456 records remained for title and abstract screening. Of these, 39 full-text reports were assessed for eligibility, and 14 studies were included from the database search. Supplementary citation-based searching and targeted identification procedures yielded three additional eligible articles, resulting in a final set of 17 included studies (**Figure 1**).

**Figure 1:**
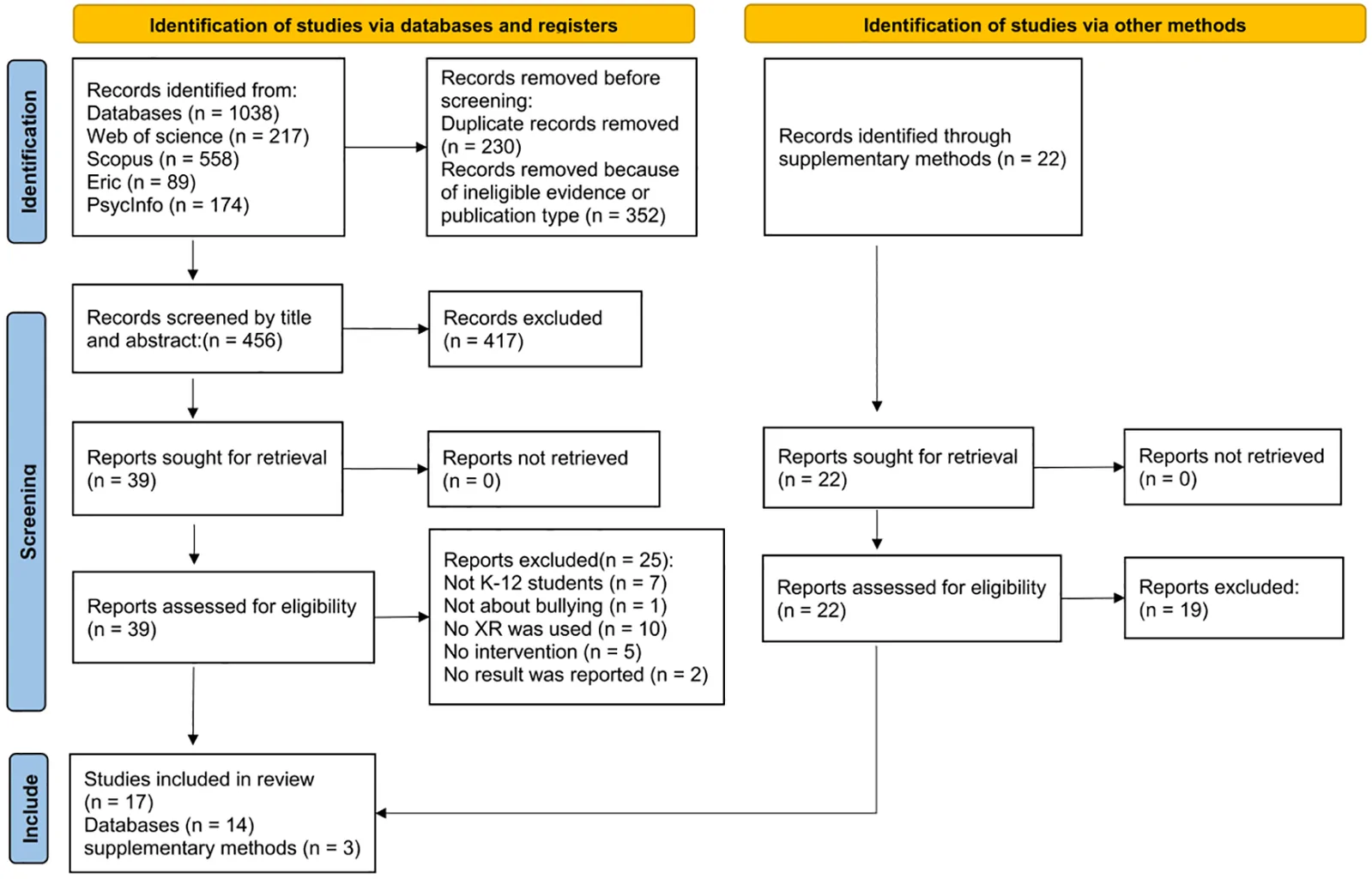
PRISMA-ScR flow diagram of the study selection process.

### 2.5 Data charting and coding

A standardized charting form was developed from the review questions and refined during the review process. It covered five main areas: basic study information, participant characteristics, XR intervention characteristics, design, and outcomes. Two reviewers independently charted the included articles, compared their coding, revisited the original articles when discrepancies arose, and resolved disagreements through discussion. The main variables included in the coding framework are summarized in **Table 2**, while the full codebook and article-level coding table are provided in the **Supplementary Materials**.

**Table 2:** Summary of the coding framework for XR-based bullying prevention research.

Dimension	Code	Type	Description/examples
Basic study information	Publication information	Quant/Qual	Publication year, country/region, journal
	Research purpose and study design	Qual	Research purpose and study design
Participant characteristics	Sample characteristics	Quant/Qual	Sample size, educational level, age/grade, gender distribution, and participant type
XR intervention characteristics	XR technology	Qual	XR modality, device, and delivery format
	Bullying-related focus and scenario	Qual	Targeted bullying-related phenomenon and intervention scenario
	XR affordances and evidence	Qual	Immersion, presence, embodiment, interaction, perspective-taking, agency, simulation, and supporting evidence
	XR perspective	Qual	Victim, bystander, observer, role exchange, or other perspective
	Intervention duration and frequency	Quant/Qual	Duration, number of sessions, and frequency
Design	Theoretical perspectives and learning objectives	Qual	Reported theoretical perspectives and intended learning goals
	Learning activities, assessment, and scaffolds	Qual	Role-play, decision making, discussion, reflection, feedback, prompts, assessment, and other support
	XR design and design logic	Qual	XR design features and reported links between theory and XR design
Outcomes	Outcome domains and measurement	Qual	Outcome domains and corresponding measures
	Findings and follow-up	Quant/Qual	Direction/statistical findings and longitudinal evidence
	Context and conclusions	Qual	Moderating/contextual factors, key conclusions, and relevant notes
Derived analytic classifications	Immersion	Qual	High or low immersion
	Interactivity	Qual	High or low interactivity
	Study function	Qual	program evaluation, mechanism-oriented research, or design/feasibility research
	Position of XR	Qual	Stand-alone activity, repeated core environment, or embedded within a broader program

For the manuscript-level synthesis, several additional analytic classifications and quantitative indicators were developed from the charted data. The immersion and interactivity scores were derived from predefined coding indicators developed for this review. Each included study was rated on each relevant indicator using a five-point scale, and composite immersion and interactivity scores were calculated by averaging the corresponding indicator scores. Two reviewers independently coded these indicators according to the predefined criteria, and disagreements were resolved through discussion. Detailed coding rubrics for immersion and interactivity scores are provided in **Supplementary Materials**. The immersion score reflected the extent to which the XR experience enhanced perceptual involvement and reduced awareness of the physical environment, whereas the interactivity score reflected the extent to which learner actions influenced the learning process, including task progression, feedback, consequences, and opportunities for response revision. Higher composite scores indicated greater levels of immersion or interactivity. Based on the composite scores, studies with scores above 3 were classified as relatively high immersion/interactivity, whereas those with scores of 3 or below were classified as relatively low immersion/interactivity for visualization in **Figure 2**. The position of XR within the intervention was classified as stand-alone activity, repeated core environment, or embedded within a broader program. Articles were also classified by their primary function as program evaluation, mechanism-oriented research, or design/feasibility research.

**Figure 2:**
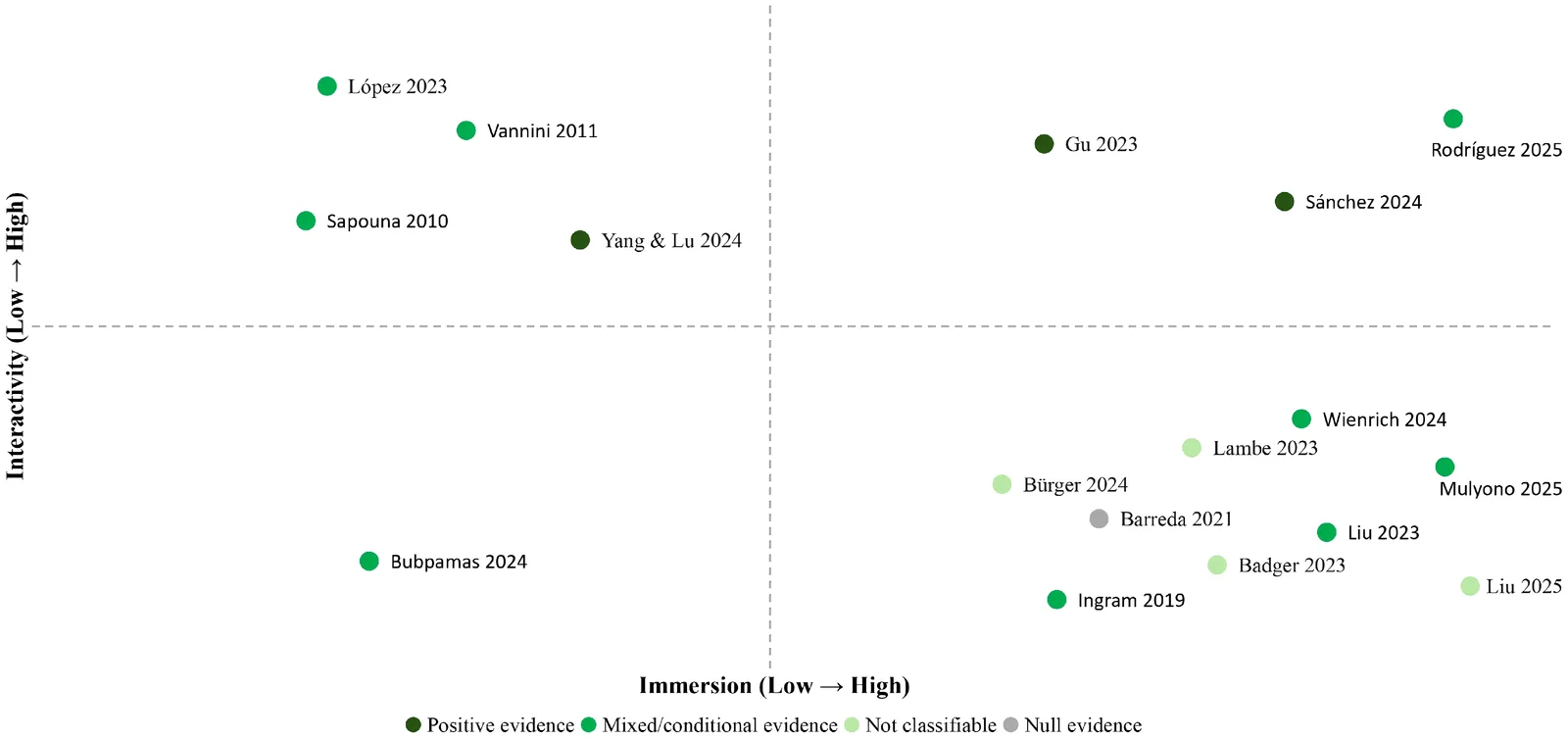
XR configurations and reported intervention-related evidence patterns. Each point represents one included article. The horizontal axis indicates immersion level, and the vertical axis indicates interactivity level. Immersion and interactivity were classified according to the definitions provided in Section 2.5. Point colors represent the primary pattern of reported intervention-related evidence. Positive evidence indicates that the study reported statistically significant or clearly described favorable changes in the selected intervention-related outcome. Mixed/conditional evidence indicates that favorable findings were observed only for some outcomes, groups, time points, or conditions. Null evidence indicates that no clear intervention-related advantage was reported. Not classifiable indicates that the study primarily examined mechanisms, design feasibility, experiential responses, or other outcomes that did not allow evaluation of intervention-related change. These classifications are descriptive and should not be interpreted as comparisons of XR effectiveness or effect magnitude across configurations.

### 2.6 Data analysis and synthesis

Categorical data were summarized using frequencies and percentages, while continuous and textual information were synthesized descriptively. The analysis first mapped publication, participant, technology, and intervention characteristics. It then examined immersion and interactivity as separate design dimensions and considered the position of XR within the wider intervention. Theoretical perspectives, XR affordances, learning activities, and outcomes were linked through qualitative content analysis. Outcome findings were organized by the type of change assessed: experiential, acceptability, and feasibility outcomes; cognitive, affective, and attitudinal outcomes; behavioral intentions, simulated actions, and real-world outcomes.

For the immersion–interactivity synthesis, each article was mapped according to two design dimensions: immersion and interactivity. High immersion referred to head-mounted or immersive 360° presentation that perceptually surrounded the learner, whereas low immersion referred to desktop virtual environments or handheld/mobile AR that retained awareness of the physical surroundings. High interactivity required learner input to influence task progression, subsequent events, feedback, consequences, or opportunities for response revision; observation, navigation, or response expression without such effects was classified as low interactivity.

To examine patterns of intervention-related findings across XR configurations, one outcome was selected for each article: the primary intervention outcome when clearly identified, or otherwise the bullying-related outcome closest to behavioral change. The primary outcome was determined based on the stated research objective, research questions, and outcome emphasized in the authors’ interpretation of findings rather than statistical significance alone. This approach was adopted to avoid assigning multiple evidence categories to studies reporting heterogeneous outcomes and to enable descriptive comparison across XR configurations. Experiential outcomes such as presence, usability, and immediate emotional or physiological responses were not used to determine intervention-related findings unless they were directly connected to the intended prevention mechanism. Findings were then classified descriptively as positive, mixed or conditional, null, or not classifiable. Positive evidence referred to statistically significant or clearly reported improvements in the target outcome; mixed or conditional evidence referred to selective, inconsistent, subgroup-dependent, or context-dependent findings; null evidence referred to no meaningful difference or change; and not classifiable evidence referred to studies that did not directly assess intervention-related outcomes. These categories were used to summarize reported patterns across studies and do not represent study quality, causal strength, or comparable effect magnitude. Because the included studies differed substantially in research purpose, intervention structure, outcome measures, and statistical reporting, the immersion–interactivity mapping was interpreted as a descriptive synthesis rather than an estimate or comparison of XR effectiveness.

## 3 Results

### 3.1 Selection and characteristics of included articles

Seventeen peer-reviewed articles published between 2010 and 2025 were included. Thirteen (76.5%) appeared from 2023 through 2025, indicating rapid recent growth. Nine articles were conducted in Europe (52.9%), six in Asia (35.3%), and two in North America (11.8%). Spain was represented in four articles and Taiwan in three. Among the K–12 samples represented in the included articles, ten focused exclusively on middle-school students, four on primary-school students, one on kindergarten children, and two included mixed educational levels. Most recruited general student samples, although several included or analyzed students according to victimization, perpetration, or non-involvement status.

The 17 articles should not be interpreted as 17 independent programs. FearNot!, TooCloseVR, and Virtual-PRO each generated related articles, and some sources shared programs or participant cohorts. Nine articles primarily evaluated prevention programs, five focused on prevention-relevant mechanisms or comparative exposure, and three emphasized system, scenario, or feasibility validation. These categories represent different research purposes and provide different types of evidence rather than equivalent evaluations of intervention effectiveness. program evaluation studies examined changes following implemented prevention interventions, whereas mechanism-oriented and design/feasibility studies focused on explaining how XR-based approaches may work, learners’ experiences with these applications, their usability and acceptability, or their initial development and validation. The main characteristics and analytic classifications of the included articles are summarized in **Table 3**.

**Table 3:** Characteristics and analytic classification of included articles.

Study	Region/educational level	N	XR configuration	Prevention focus	Study purpose	XR position	Follow-up
Badger et al. (19)	United Kingdom; Middle school	67	Immersive VR	Traditional bullying	Mechanism-oriented study	Stand-alone activity	None
Barreda-Ángeles et al. (20)	Spain; Primary school	35	360° VR video	Traditional bullying	Design/feasibility study	Stand-alone activity	None
Bürger and Wienrich (21)	Germany; Middle school	92	Immersive VR	Cyberbullying	Design/feasibility study	Embedded within broader program	1 h
Gu et al. (22)	China; Middle/high school	214	Embodied immersive VR	Traditional bullying	Mechanism-oriented study	Stand-alone activity	None
Ingram et al. (23)	United States; Middle school	118	Mobile 360° VR	Traditional/cyberbullying and relational aggression	program evaluation study	Embedded within broader program	None
Lambe and Craig (24)	Canada; Upper primary/middle school	120	Immersive VR	Peer victimization/social exclusion	Mechanism-oriented study	Stand-alone activity	None
Liu et al. (25)	Taiwan; Middle school	229	Immersive VR	Traditional bullying	Mechanism-oriented study	Stand-alone activity	None
Liu et al. (26)	Taiwan; Middle school	209	Immersive VR	Traditional bullying	Mechanism-oriented study	Stand-alone activity	None
López-Faican and Jaen (27)	Spain; Middle school	30	Mobile AR and geolocation	Bullying within broader helping scenarios	program evaluation study	Repeated core environment	None
Mulyono et al. (28)	Indonesia; Middle school	98	Immersive VR	Traditional bullying	program evaluation study	Repeated core environment	None
Sapouna et al. (29)	United Kingdom/Germany; Primary school	942	Desktop virtual environment	Traditional bullying	program evaluation study	Repeated core environment	1 and 4 weeks
Wienrich et al. (30)	Germany; Middle school	92	Immersive VR	Cyberbullying	Design/feasibility study	Embedded within broader program	1 h
Bubpamas et al. (31)	Thailand; Kindergarten	90 children; 9 teachers	Mobile AR storybooks	Traditional bullying	program evaluation study	Embedded within broader program	None
Vannini et al. (32)	United Kingdom/Germany; Primary school	770 non-involved subsample	Desktop virtual environment	Traditional bullying/defending	program evaluation study	Repeated core environment	During intervention + 4 weeks
Sánchez-Jiménez et al. (33)	Spain; Middle school	579 at baseline	Interactive 360° VR	Peer sexual harassment	program evaluation study	Embedded within broader program	3 months
Yang and Lu (34)	Taiwan; Primary school	56	Desktop role-playing environment	Traditional and cyberbullying	program evaluation study	Repeated core environment	None
Rodríguez-de Arriba et al. (35)	Spain; Middle school	652 complete cases	Interactive 360° VR versus 2D	Peer sexual harassment	program evaluation study	Embedded within broader program	3 months

### 3.2 XR configurations, interactivity, and evidence patterns

VR and virtual environments dominated the evidence base, but immersion and interactivity represented distinct rather than parallel design dimensions. Fifteen articles (88.2%) used VR or virtual environments, whereas two used AR. Twelve articles adopted high-immersion configurations involving head-mounted displays or immersive 360° presentation, and five used lower-immersion desktop or mobile AR configurations. Nine articles combined high immersion with low interactivity, three combined high immersion with high interactivity, four combined low immersion with high interactivity, and one combined low immersion with low interactivity (**Figure 2**). Thus, high immersion did not necessarily involve meaningful learner interaction, whereas several lower-immersion systems incorporated learner decisions, feedback, or opportunities for response revision as central components of the activity.

The distribution of reported intervention-related evidence patterns varied across immersion–interactivity configurations. Studies classified as high interactivity included several articles reporting favorable or conditionally favorable evidence patterns, whereas studies classified as low interactivity included a wider range of reported evidence patterns, including mixed, null, and not classifiable findings. Among high-interactivity studies, positive or conditionally positive findings were reported in both high-immersion and lower-immersion systems, including studies involving decision making, feedback, consequence visualization, or response revision. By contrast, several low-interactivity studies primarily reported changes related to experiential, psychological, or mechanism-oriented outcomes rather than direct intervention-related changes. Overall, the distribution indicates that studies with different immersion–interactivity configurations examined different types of outcomes and reported varied evidence patterns.

The two AR studies further illustrate differences in how interaction was incorporated into XR-based bullying-related activities. López-Faican and Jaen (27) used location-based AR helping activities that required learners to complete actions within the environment and was classified as high interactivity. In contrast, Bubpamas et al. (31) used AR storybooks combined with teacher-mediated questioning and judgment activities and was classified as low interactivity. Although Bubpamas et al. (31) reported pre–post changes following the intervention, the study design primarily evaluated changes within the implemented learning activity.

High-immersion, low-interactivity designs primarily used XR as an experiential medium for exposure, perspective-taking, and emotional engagement rather than as an environment for extended decision practice. These studies typically presented scripted bullying, exclusion, or harassment scenarios from victim or bystander perspectives. Learners could explore the environment, experience embodied perspectives, or express responses, but generally had limited influence over subsequent events. Badger et al. (19)Barreda-Ángeles et al. (20), and Liu et al. (25, 26 examined psychological, emotional, or bystander-related responses within these scenarios, although their research purposes and outcome measures differed. Ingram et al. (23) embedded relatively linear VR scenarios within a broader curriculum, whereas Bürger and Wienrich (21) and Wienrich et al. (30) used restricted learner control in TooCloseVR scenarios.

High-interactivity designs differed by incorporating learner actions that influenced subsequent events, feedback, or consequences within the activity process. Among the high-immersion, high-interactivity studies, Gu et al. (22) combined embodiment, tracked actions, and bully–victim role exchange, whereas Sánchez-Jiménez et al. (33) and Rodríguez-de Arriba et al. (35) incorporated decision-making within immersive victim and bystander scenarios. Lower-immersion systems also supported learner interaction without immersive displays. Sapouna et al. (29) and Vannini et al. (32) used FearNot! to allow learners to advise virtual victims and observe subsequent story developments. López-Faican and Jaen (27) linked location-based actions to helping tasks, and Yang and Lu (34) organized learning around repeated choices, feedback, and response revision. Together, these studies suggest that immersion and interactivity represent separable design dimensions with diverse combinations across XR-based bullying prevention interventions.

### 3.3 Intervention structure and instructional design

#### 3.3.1 Position of XR within the intervention

XR served three recurring functions within bullying-related interventions: stand-alone experiential or experimental experience, repeated interactive practice environment, and embedded instructional component. Six articles used XR primarily as a brief stand-alone experience or core experimental component (19, 20, 22, 24–26). These studies mainly examined learners’ immediate experiences, perceptions, emotional responses, or responses within controlled scenarios rather than positioning XR as a complete instructional program.

Five articles used virtual or augmented environments repeatedly to support coping, helping, emotion-regulation, or problem-solving practice (27–29, 32, 34). In these studies, XR functioned as a space for learners to make decisions, observe consequences, practice responses, or revise strategies. For example, Sapouna et al. (29) and Vannini et al. (32) used FearNot! to allow learners to observe successive bullying episodes, advise virtual victims, and observe how their suggestions influenced subsequent events.

Six articles embedded XR within a broader workshop or curriculum containing instruction, discussion, reflection, role-play, or other complementary activities (21, 23, 30, 31, 33, 35). In these studies, XR functioned as one component within a larger instructional sequence rather than as an independent intervention mechanism. The same technology label therefore represented substantially different intervention structures, with XR serving different functions depending on whether it was used for experience, practice, or integration within broader learning activities.

#### 3.3.2 Theory–affordance–activity alignment

Theoretical perspectives and intervention rationales were diverse and often combined, but three recurring mechanism orientations were identifiable across the included studies. First, perspective-taking and experiential approaches used first-person experience, role change, or multiple viewpoints to help learners understand bullying situations from different social positions. Gu et al. (22) used embodied role exchange between bully and victim positions to create opportunities for learners to examine bullying from different social perspectives, whereas Yang and Lu (34) combined multiple roles with branching consequences and opportunities for response revision. Second, bystander-intervention and responsibility-taking approaches focused on recognizing harm, taking responsibility, considering possible responses, and developing intentions or strategies for intervention. This orientation was evident in Sánchez-Jiménez et al. (33) and Rodríguez-de Arriba et al. (35), whereas Liu et al. (26) examined factors associated with bystander behavior with moral courage as a key explanatory construct. Third, coping and social-learning approaches emphasized response strategies and learning from observed consequences. This perspective was particularly evident in Sapouna et al. (29) and Vannini et al. (32), where learners advised virtual victims and observed how their suggestions influenced subsequent story developments.

Several studies also incorporated more specific theoretical perspectives or learning principles to explain how XR-based activities were designed. Ingram et al. (23), for example, explicitly drew on Construal Level Theory and designed VR experiences to reduce the perceived psychological distance of bullying situations through perspective-taking and reflection. Bürger and Wienrich (21) and Wienrich et al. (30) similarly used immersive scenarios designed to make cyberbullying experiences more immediate and personally relevant, although they did not explicitly frame their designs through Construal Level Theory. Mulyono et al. (28) developed a VR-based emotion-regulation intervention grounded in self-awareness and emotional processing, using guided relaxation, emotional visualization, forgiveness exercises, and acceptance-oriented activities to address bullying-related experiences. Across studies, XR features were used in different ways: immersion was used to increase the experiential immediacy of bullying-related situations, role enactment was used to support perspective-taking, and interactive choices and feedback were used to support coping-related reflection, decision making, and bystander-response practice. These recurring links among theoretical mechanisms, XR affordances, learning activities, and outcomes are summarized in **Table 4**.

**Table 4:** Recurring links among mechanisms, XR affordances, activities, and outcomes.

Theoretical mechanism/learning rationale	XR affordance	Learning activity	Proximal outcomes examined	Illustrative studies
Perspective-taking and empathic understanding	First-person perspective; embodiment; role change; presence	Experience bullying situations from different social positions; compare victim, bystander, and other perspectives; reflect on experiences	Understanding of bullying dynamics; empathy-related responses; recognition of others’ experiences	Gu et al. (22)Ingram et al. (23)Yang and Lu (34)
Psychological proximity and emotional engagement	Immersion; realistic social scenarios; spatial presence	Experience bullying or cyberbullying situations designed to increase immediacy and emotional involvement	Presence; perceived realism; emotional responses; perceived relevance of bullying situations	Barreda-Ángeles et al. (20)Bürger and Wienrich (21)Wienrich et al. (30)
Bystander decision making and intervention preparation	Scenario simulation; decision points; role-based choices	Identify harmful situations; evaluate possible responses; practice intervention-related decisions	Intervention intentions; responsibility judgments; simulated response choices	Liu et al. (26)Sánchez-Jiménez et al. (33)Rodríguez-de Arriba et al. (35)
Coping and social learning through consequences	Virtual agents; interactive narratives; consequence visibility	Provide advice, observe consequences, and consider alternative coping or support strategies	Coping strategies; defending-related intentions; understanding of response consequences	Sapouna et al. (29)Vannini et al. (32)
Agency, feedback, and response revision	Branching choices; feedback; consequence visibility; response revision opportunities	Make decisions, receive feedback, revise ineffective responses, and compare alternative strategies	Learning achievement; problem-solving tendencies; strategy selection	Yang and Lu (34); FearNot!-based studies

These mechanism orientations were reflected in two broad instructional patterns that represent different ways of organizing learning processes rather than different levels of intervention effectiveness. In the experience–reflection pattern, XR provided a bullying-related experience that was subsequently interpreted through discussion, writing, role-play, media production, or other reflective activities. Bürger and Wienrich (21)Ingram et al. (23)Wienrich et al. (30)Sánchez-Jiménez et al. (33), and Rodríguez-de Arriba et al. (35) followed this general pattern, with much of the interpretation and strategy development occurring around the XR experience. In the action–feedback–revision pattern, more of the learning process occurred within the digital environment itself. Sapouna et al. (29) and Vannini et al. (32) allowed learners to advise virtual victims and observe how their suggestions influenced subsequent events, whereas Yang and Lu (34) enabled learners to select responses, receive feedback or hints, and revise ineffective strategies. These two patterns differed mainly in the location of reflection, feedback, and strategy development—around the XR experience or within the digital environment—and should not be interpreted as evidence that one pattern is superior to the other.

### 3.4 Reported outcomes and evidence patterns

#### 3.4.1 Experiential, acceptability, and feasibility outcomes

The most consistent findings concerned learners’ immediate experiences and the acceptability or feasibility of XR-based activities. Immersive conditions frequently produced stronger presence, perceived realism, physiological arousal, tension, or negative affect than neutral or screen-based conditions. Badger et al. (19) reported higher negative affect after exposure to a hostile virtual classroom, while Barreda-Ángeles et al. (20) found greater presence and physiological arousal in VR than during screen viewing of matched 360° scenes. Bürger and Wienrich (21) and Wienrich et al. (30) reported increased tension and feelings of intrusion or constriction immediately after exposure, with these responses moving back toward baseline within the following hour. Usability and satisfaction were generally favorable where assessed, and severe simulator sickness or attrition attributable to discomfort was uncommon. Reported challenges mainly involved dizziness, headset weight, audiovisual quality, device cost, and software stability.

#### 3.4.2 Cognitive, affective, and attitudinal outcomes

Cognitive, affective, and attitudinal outcomes were among the most frequently examined outcomes in XR-based bullying-related studies. These outcomes primarily concerned learners’ understanding of bullying situations, perspective-taking, empathy-related responses, emotional engagement, and attitudes toward bullying or intervention, rather than direct changes in real-world bullying behavior. Across studies, XR was commonly used to provide experiential contexts that made social situations more accessible, vivid, and controllable and allowed learners to examine bullying from different perspectives, although reported outcomes varied across measures, populations, and study designs.

Cognitive outcomes included bullying recognition, learning achievement, strategy recall, and understanding of appropriate responses. Several studies examined these outcomes with different measures and comparison conditions. Barreda-Ángeles et al. (20) showed that participants could distinguish bullying-related scenes from neutral scenes, but VR did not improve recognition relative to screen viewing. Yang and Lu (34) reported higher anti-bullying learning scores for multi-role than single-role virtual learning. Wienrich et al. (30) found that students demonstrated understanding of the intended “no escape” message of the cyberbullying scenario during post-experience discussions and reported understanding of self-help and bystander strategies. Overall, cognitive findings varied according to intervention design and evaluation approach, with no consistent advantage observed for higher immersion alone.

Affective and attitudinal outcomes were more frequently examined, particularly in studies incorporating role-taking, perspective-based experiences, or bystander scenarios. Empathy-related findings varied across cognitive, affective, and distress-related components. Improvements were reported in several studies involving role exchange, multi-role learning, AR helping activities, and VR-enhanced curricula (22, 23, 27, 34). For example, Ingram et al. (23) reported higher empathy in the intervention group, while Yang and Lu (34) found higher empathy in the multi-role condition. However, empathy-related changes were not consistently observed across studies. Liu et al. (25) found that VR exposure produced stronger emotional responses, including fear and excitement, but lower empathy and defender self-efficacy than the text condition. Wienrich et al. (30) reported changes in cognitive media-based empathy, whereas affective empathy outcomes were less consistent. Rodríguez-de Arriba et al. (35) and Sánchez-Jiménez et al. (33) reported selected changes in moral disengagement, hostile sexism, and intervention-related attitudes within the Virtual-PRO program, but the VR version did not consistently outperform the content-equivalent 2D program.

#### 3.4.3 Behavioral intentions, simulated actions, and real-world outcomes

Most included articles reported at least one behavioral or behavior-related outcome. These outcomes varied in their proximity to real-world bullying behavior and included behavioral intentions, responses within simulated environments, and reported changes in bullying-related behaviors. Behavioral intentions included willingness to defend, support a victim, intervene as a bystander, or refrain from reinforcing bullying or remaining passive as outsiders. Positive findings were reported in some role-exchange, bystander, and curriculum studies across different types of behavioral evidence, while null or conditional findings were also common. Among the measured prevention outcomes, Rodríguez-de Arriba et al. (35) found that willingness to intervene for a non-friend was the only outcome for which the VR version showed a significant advantage over the non-VR version, although the magnitude of this difference was small (d = 0.07).

Simulated actions provided a different form of behavioral evidence. Lambe and Craig (24) recorded comforting and solution-focused responses during virtual exclusion, with 33% of participants displaying at least one defending behavior. Yang and Lu (34) recorded learners’ actions after incorrect responses, including hint seeking, further answering, and revision toward an appropriate solution. Rodríguez-de Arriba et al. (35) also recorded choices made within interactive scenarios and found differences in victim-response choices, with VR participants more often selecting the option of talking with family members. These measures capture what learners did within structured scenarios, but their responses remained shaped by the options and conditions built into the system. They therefore provide evidence of behavior within simulation rather than direct evidence of how students would respond during real bullying situations.

Evidence of sustained change in bullying-related behavior was more limited. Sapouna et al. (29) found that students identified as victims at baseline showed a greater likelihood of no longer being classified as victims at the one-week follow-up, but this effect was not maintained at four weeks and differed between countries. Vannini et al. (32) also reported country-dependent changes in defender emergence. Sánchez-Jiménez et al. (33) and Rodríguez-de Arriba et al. (35) reported selected changes in bystander-related and victimization-related outcomes at three months, although these findings were not consistent across all measures or intervention formats. Overall, four articles followed K–12 participants for at least one week, and only two extended follow-up to three months, the longest follow-up period reported among the included K–12 studies. Behavioral evidence ranged from intentions and actions within simulated environments to self-, peer-, or teacher-reported changes in bullying-related behavior, but evidence of sustained change over time remained limited.

## 4 Discussion

This review mapped 17 articles on XR-based bullying prevention interventions for K–12 students and identified a field that is growing rapidly but remains conceptually and methodologically heterogeneous. Three findings organize the evidence. First, high immersion was usually paired with limited learner control, whereas lower-immersion systems more often emphasized choices, feedback, and revision. Second, the instructional function of XR depended on its position within a broader design: some applications delivered a salient experience for later reflection, while others embedded response practice and feedback within the digital environment. Third, experiential outcomes were more consistent than intervention outcomes, and evidence weakened as outcomes moved from immediate reactions toward sustained real-world behavior.

### 4.1 Immersion, interactivity, and intervention outcomes

The distinction between experiential intensity and intervention-related change provides an important lens for interpreting XR-based bullying prevention research. Across the reviewed studies, immersive configurations consistently strengthened experiential responses, including presence, realism, physiological arousal, emotional engagement, or perceived intrusion, whereas findings for bullying recognition, empathy, self-efficacy, attitudes, and behavioral responses were less consistent. This distinction is consistent with research showing that technological immersion can strengthen presence (36) and with the Cognitive Affective Model of Immersive Learning, which conceptualizes presence and agency as processes through which immersive environments may shape subsequent affective, cognitive, and motivational processes (37). In bullying prevention, immersion can therefore make a situation more immediate, salient, and personally involving, but a stronger experience does not by itself determine what learners understand or how they respond afterward.

These findings highlight that different XR affordances may contribute to different stages of the intervention process. Immersion primarily changes how learners encounter a bullying-related situation, whereas interactivity changes the extent to which learners can act within that situation, receive consequences, and revise responses. Many high-immersion, low-interactivity studies presented vivid but largely predetermined scenarios. These designs were well suited to eliciting presence, emotional responses, psychological proximity, or perspective experience, but they offered limited opportunities for learners to test responses, observe consequences, or refine strategies. By contrast, several studies incorporating meaningful learner interaction reported favorable or conditionally favorable evidence patterns, including several lower-immersion desktop and AR systems in which learner decisions, feedback, and response revision were central components of the activity. This pattern suggests that interactivity may represent an important design dimension for bullying prevention, particularly when interventions aim to support response selection, coping, or behavioral rehearsal. However, this finding should be interpreted as a descriptive association rather than evidence that interactivity alone produces stronger prevention effects. Highly interactive systems were also more often evaluated as complete learning interventions, whereas several lower-interactivity studies primarily examined experiential responses, psychological processes, or mechanism-related outcomes.

The few studies that directly compared immersive and less immersive presentations reinforce this distinction between experiential intensity and intervention value. Barreda-Ángeles et al. (20) found greater presence in VR without a corresponding advantage in bullying recognition, while Liu et al. (25) reported stronger emotional responses in VR but no consistent advantage for empathy or defender self-efficacy. Similarly, the content-equivalent VR and 2D versions of Virtual-PRO produced broadly comparable outcomes, with only selective advantages for the VR condition on proximal outcomes (35). Taken together, these findings indicate that increasing immersion can strengthen the experience of a bullying situation without necessarily strengthening the cognitive, attitudinal, or behavioral changes that follow. For prevention design, the more useful question is therefore not simply how immersive or interactive an XR system is, but what specific features enable learners to experience, decide, and practice in relation to the intended intervention goal.

### 4.2 Learner agency and role participation

The educational value of interaction depends less on the number of available actions than on the degree of learner agency created through those actions. Simple interactions, such as looking around, selecting an option, or expressing a response, provide limited agency when they do not influence subsequent events or learning processes. Agency becomes more meaningful when learners’ decisions affect what happens next, generate feedback, reveal consequences, or create opportunities to revise earlier responses. Sapouna et al. (29) and Vannini et al. (32), for example, allowed learners to advise virtual victims and observe how their suggestions influenced later events, whereas Yang and Lu (34) enabled learners to make choices, receive feedback, and revise responses. These designs created a learning process in which students could act on a situation, observe the consequences of their decisions, and reconsider alternative strategies. For bullying prevention, interaction may therefore be particularly valuable when it supports situated decision making rather than simply increasing the number of available actions.

Learner roles further shape how agency is exercised because bullying involves different social positions with distinct information, responsibilities, and possible responses. As bullying is embedded within peer-group dynamics, victims, bystanders, defenders, and perpetrators may interpret the same event differently and face different behavioral choices (5). A victim role may direct attention toward experiences of harm and possible coping strategies, whereas a bystander role requires learners to recognize problematic situations, evaluate responsibility, and consider whether and how to intervene. Role changes can extend this process by requiring learners to reconsider the same event from alternative social positions. Gu et al. (22), for example, used bully–victim role exchange, while Yang and Lu (34) allowed learners to experience multiple roles within a virtual environment. In these designs, role participation changes not only what learners see but also what they are expected to notice, judge, and do.

Agency and role participation should therefore be considered together. Agency determines the extent to which learners can influence the unfolding situation, whereas role participation determines the perspective and responsibilities through which decisions are made. Their combination may be particularly relevant for bullying prevention because responding to bullying requires learners to interpret situations from a particular social position and determine appropriate actions within that context. However, increasing agency or adding more roles does not automatically improve learning. Perpetrator roles, emotionally intense victim experiences, or complex decision environments may create additional cognitive and emotional demands, particularly for younger learners. Role selection and interaction design should therefore be aligned with the intended learning goal, developmental level, and support provided during and after the XR experience.

### 4.3 Aligning theory, affordances, activities, and evaluation

Building on the role of agency and situated decision making, the findings suggest that the label “XR intervention” alone provides limited explanatory value unless the underlying intervention logic is specified. The same technological feature may serve different educational purposes depending on the mechanism it is intended to support, the activity structure surrounding it, and the outcomes being evaluated. For example, immersion may be used to increase the experiential immediacy of bullying situations, support perspective-taking, or examine emotional responses; interactivity may be used to facilitate decision making, response practice, or strategy revision. Therefore, XR design should begin with the intended prevention mechanism rather than with the selection of a particular technology.

A mechanism–affordance–activity–outcome alignment provides a useful framework for understanding and designing XR-based bullying prevention interventions. Theoretical mechanisms specify the psychological or behavioral processes an intervention seeks to influence, such as perspective-taking, responsibility recognition, coping, or bystander decision making. XR affordances provide ways through which these processes may be supported, including embodiment, multiple viewpoints, interactive choices, feedback, and consequence visualization. Learning activities translate these affordances into instructional experiences, such as role exchange, scenario-based decision making, reflection, or strategy rehearsal. Outcomes should then be selected according to the level of change the intervention is designed to produce, ranging from experiential responses and proximal psychological outcomes to behavioral intentions, simulated actions, and real-world behavioral indicators.

This alignment also highlights the importance of distinguishing between the XR component and the broader instructional intervention. Several included studies embedded XR within workshops, curricula, discussions, or reflective activities, indicating that XR functioned as one component of a broader instructional system rather than as an isolated intervention. The educational value of XR may therefore depend partly on how experiences are prepared, interpreted, and extended beyond the technology itself. Conversely, interventions that rely primarily on XR exposure should ensure that the measured outcomes correspond to what the experience is designed to influence. For example, a system intended to create emotional engagement may be appropriately evaluated through experiential or affective outcomes, whereas a system intended to develop coping strategies or bystander responses requires measures closer to behavioral decision making.

Future research would benefit from making these alignment decisions explicit before implementation. Rather than asking whether XR is effective in general, studies should specify which mechanism is targeted, which affordances are expected to support that mechanism, what learning activities are provided, and whether the selected outcomes capture the intended level of change. Such specification would improve theoretical interpretation, facilitate comparison across studies, and clarify when XR provides added value beyond less immersive or non-digital alternatives.

### 4.4 Outcome proximity and inferential limits

The outcomes reported in the reviewed studies reflected different levels of change. Presence, affect, and perceived realism mainly describe how learners experienced the XR environment. Knowledge, empathy, and attitudes indicate changes in understanding or psychological responses. Behavioral intentions show whether learners are willing to act, while simulated choices show how they respond within a designed situation. These outcomes are related, but they should not be treated as the same type of evidence. A student may report a stronger intention to defend a victim or choose an appropriate response in a virtual scenario, but this does not necessarily mean that the student will act in the same way during a real bullying event. Real peer situations involve social relationships, group pressure, and possible consequences that are difficult to reproduce fully in a simulation. Simulated behavior can therefore show whether learners are able to apply a response in a controlled setting, but it cannot by itself demonstrate change in everyday bullying behavior. Conclusions about intervention effects should match the type of outcome that was actually measured.

Evidence became more limited when the focus moved from immediate responses to changes in real-world behavior over time. Although many of the included articles reported at least one behavioral or closely related outcome, many measured intentions or responses within simulated situations rather than actual changes in bullying, victimization, or defending behavior. The available follow-up findings also suggest that change may develop differently over time. Sapouna et al. (29), for example, found that students identified as victims at baseline were more likely to no longer be classified as victims at the one-week follow-up, but this effect was not maintained at four weeks and differed between countries. Sánchez-Jiménez et al. (33) and Rodríguez-de Arriba et al. (35) reported some changes in bystander-related and victimization-related outcomes at three months, but these changes were not consistent across all measures. Follow-up is therefore important not simply to determine whether an immediate effect remains, but to examine whether early changes disappear, continue, or lead to later changes in behavior. Future studies should track this process more directly by examining whether immediate changes in experience, knowledge, attitudes, or intentions are followed by changes in how students respond to bullying in everyday settings.

### 4.5 Implications for research and practice

For researchers, future work should report XR interventions in greater detail rather than relying on broad labels such as “VR intervention.” At minimum, studies should specify immersion, interactivity, learner agency, learner roles, and the position of XR within the intervention. When XR is combined with other teaching activities, researchers should make clear which part of the intervention is expected to produce each outcome and, where possible, examine the specific contribution of XR. Outcome measures should also match the intended level of change. Studies involving children should report preparation, safeguarding, and debriefing procedures, particularly when emotionally intense scenarios or sensitive roles are used.

For schools, XR should be selected according to the learning goal rather than the level of immersion alone. A useful starting point is to determine whether students mainly need to experience a bullying situation, practice how to respond to it, or combine both forms of learning, and then select the XR format and accompanying activities that support that purpose. The technology should therefore follow the instructional design, rather than determine it. Developmental appropriateness, teacher guidance, and opportunities for reflection should also be considered when XR is introduced into bullying-prevention activities.

### 4.6 Limitations

This review has several limitations. First, only peer-reviewed journal articles published from 2005 through 2025 were included. Conference proceedings, dissertations, and other gray literature were excluded, although emerging XR applications may first appear in these sources. The review may therefore underrepresent early-stage systems and unpublished findings.

Second, although formal title and abstract screening and full-text assessment were independently conducted by two reviewers, the preliminary screening of records that were clearly unrelated to the review scope or did not meet predefined evidence-type or publication-type criteria was conducted by the first reviewer alone. Therefore, the possibility remains that some potentially relevant records may have been excluded during this initial efficiency-oriented screening step.

Third, the review included studies at different stages of intervention development, ranging from mechanism and scenario testing to full program evaluation. These studies addressed different research questions and therefore provide different kinds of evidence. Findings from mechanism or feasibility studies should not be interpreted in the same way as findings from studies designed to evaluate intervention outcomes.

Fourth, several articles examined the same program or overlapping participant samples, and the included studies varied substantially in technology, design, comparison condition, outcomes, and follow-up. The frequencies and immersion–interactivity patterns reported in this review therefore describe articles rather than fully independent interventions or participant samples. These patterns should be interpreted as characteristics of the available literature rather than as estimates of how often particular designs are effective.

Finally, no formal quality appraisal or risk-of-bias assessment was conducted. This is consistent with the mapping purpose of the review, but it limits conclusions about the strength of individual studies. The review can identify recurring design patterns, reported outcomes, and gaps in the literature, but it cannot determine overall intervention effectiveness or whether particular XR features directly cause better outcomes.

## 5 Conclusion

XR-based bullying prevention research among K–12 students has moved beyond simple technology demonstrations toward a diverse set of experiential, role-based, and curriculum-integrated designs. Its clearest strength is the capacity to make bullying situations controlled, repeatable, salient, and accessible from perspectives that are difficult to reproduce safely in classrooms. However, immersion most consistently influenced how learners experienced bullying-related situations rather than the prevention outcomes that followed. Across the reviewed studies, several designs incorporating meaningful learner interaction reported favorable or conditionally favorable intervention-related evidence patterns. This descriptive pattern suggests that opportunities for decision making, feedback, and response revision may represent potentially relevant components of XR-based prevention design. Progress will depend on treating XR as one component of an intervention system rather than as the intervention itself. The most informative future designs will specify the targeted mechanism, select XR affordances and learning activities accordingly, connect experiences to appropriate forms of reflection or feedback, and evaluate outcomes at the level and timescale claimed. Such work can clarify when XR adds meaningful prevention value, when lower-cost formats are sufficient, and how digitally mediated experiences can contribute to safer peer environments.
