# Supplementary material for: XR-based bullying prevention interventions among K–12 students: a scoping review of design, theory, and outcomes
Source: Front Psychiatry. 2026 Oct 6;17:1978530. doi: 10.3389/fpsyt.2026.1978530 (PMC13681256; doi:10.3389/fpsyt.2026.1978530)
Supplement: Supplementary file 1 [file DataSheet1.pdf]

Coding rubric for immersion and interactivity scores

A. Immersion coding rubric

Score	Coding criteria
1	The learning experience provides minimal perceptual involvement with limited XR-related experiential features. Learners mainly receive information through conventional screen-based or passive presentation formats.
2	The experience provides limited environmental engagement. Learners may interact with a digital representation, but the environment does not substantially surround or involve the learner.
3	The experience provides moderate immersion through enhanced visual, spatial, or embodied features, but learners maintain substantial awareness of the physical environment.
4	The experience provides a strong sense of environmental presence through immersive displays, 360° environments, or embodied interaction, reducing awareness of the physical surroundings.
5	The experience provides highly immersive engagement through multiple XR affordances, including strong environmental enclosure, embodiment, and sustained perceptual involvement.

B. Interactivity coding rubric

Score	Coding criteria
1	Learners mainly observe or experience a predefined scenario without meaningful input or influence on the learning process.
2	Learners have limited control, such as navigation, viewpoint adjustment, or simple responses that do not substantially affect subsequent events.
3	Learners can perform actions or make selections within the environment, but feedback or consequences are limited.
4	Learner actions influence subsequent events, feedback, or task progression, allowing learners to explore different responses.
5	Learners actively participate in decision making, receive meaningful consequences or feedback, and have opportunities to revise strategies or responses.
